# Revealing Annexin A2 and ARF-6 enrollment during *Trypanosoma cruzi* extracellular amastigote-host cell interaction

**DOI:** 10.1186/s13071-015-1097-6

**Published:** 2015-09-29

**Authors:** Thaise Lara Teixeira, Lilian Cruz, Renato Arruda Mortara, Claudio Vieira Da Silva

**Affiliations:** Instituto de Ciências Biomédicas, Universidade Federal de Uberlândia, Uberlândia, Brasil; Universidade Federal de São Paulo, São Paulo, Brazil; Laboratório de Tripanosomatídeos, Disciplina de Imunologia - Instituto de Ciências Biomédicas, Universidade Federal de Uberlândia, Rua Piauí, Bloco 2B sala 200, Campus Umuarama, Uberlândia, MG 38400-902 Brasil

**Keywords:** *Trypanosoma cruzi*, Extracellular amastigotes, Annexin A2, ARF-6, Cell invasion, Actin cytoskeleton

## Abstract

**Background:**

Invasion of host cells by *Trypanosoma cruzi* extracellular amastigotes is host actin polymerization-dependent. However, the role of proteins related to actin dynamics during invasion by amastigotes remains to be investigated. Here we describe the role of Annexin A2 and ARF-6 during extracellular amastigote-mammalian cell interactions.

**Findings:**

Our results showed ARF-6 accumulation in the amastigote-containing parasitophorous vacuole containing amastigote forms; demonstrated ARF-6 and Annexin A2 critical impact over parasite cell invasion and revealed the effect of Annexin A2 expression on intracellular parasite multiplication.

**Conclusion:**

ARF-6 and Annexin A2 are involved in invasion of mammalian cells by *T. cruzi* amastigotes.

## Findings

The Annexin family proteins are implicated in a wide range of cellular responses triggered by increased cytoplasmatic calcium levels [1]. Annexin A2 has an essential role in actin-based macropinocytic rocketing [2] and is expressed at the interface between F-actin and membranes enriched in phosphatidylinositol 4,5,-biphosphate (PIP_2_) [3]. Moreover, Annexin A2 is recruited to membrane structures enriched in F-actin during enterophatogenic *Escherichia coli* host cell adhesion [4].

The ARF family of small GTPases regulates membrane trafficking and actin cytoskeleton rearrangements [5]. ADP-ribosylation factor 6 (ARF-6) participates during *Chlamydia caviae* and *Yersinia pseudotuberculosis* host cell invasion by acting on actin cytoskeleton polymerization [6–8]. In this context, ARF-6 showed an important role during *Toxoplasma gondii* cell invasion activating PI3-kinase signaling pathway and mobilizing PIP_2_ and PIP_3_ to the parasite parasitophorous vacuole [9].

Invasion of host cells by *Trypanosoma cruzi* extracellular amastigotes (EA) is host actin polymerization-dependent. EAs induce the formation of crater or cup-like structures enriched in F-actin reliant on host cell type [10]. Here we aimed to study the role of actin polymerization-related proteins, Annexin A2 and ARF-6, during EA host cell interaction *in vitro*.

**Fig. 1:**
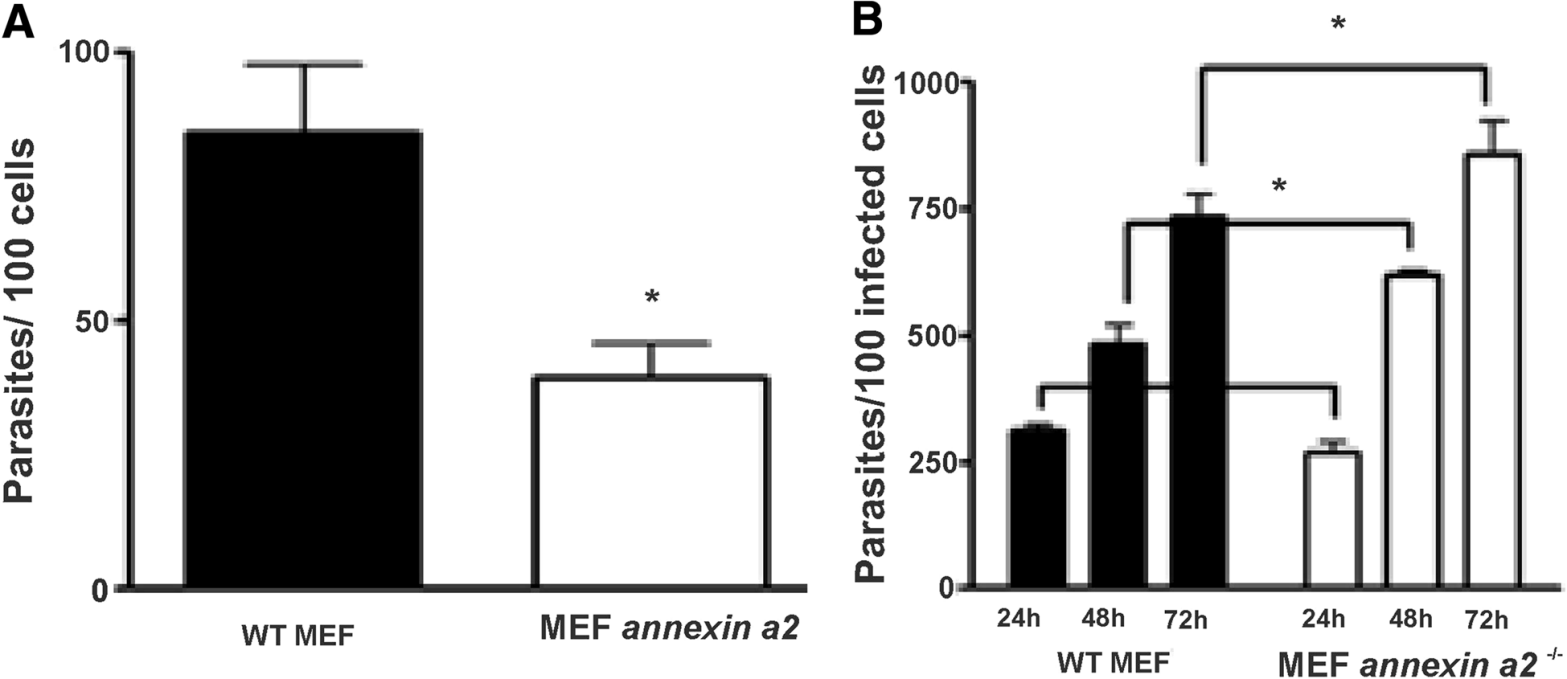
Annexin A2 expression is required during host cell invasion by *T. cruzi*. **a, b:** 5x10^4^ wild-type and Annexin A2 knockout murine embryonic fibroblasts (MEF) (a gift from Prof. Dr. Stephen E. Moss, Institute of Ophthalmology, University College London)/ well were seeded into 24 well plates overnight. Cells were infected with *T. cruzi* G-strain EAs for one hour at a multiplicity of infection (MOI) of five parasites/cell. Cells were then washed with PBS, fixed with Bouin and stained with Giemsa. The results of three independent experiments performed in triplicate are shown. The number of internalized parasites was determined by counting 200 cells/coverslip. 200 infected cells/coverslip were counted in multiplication assays. Significant differences were determined by GraphPad Prism software, version 5.01 and Student’s t-test was used. Differences were considered significant when p < 0.05

**Fig. 2:**
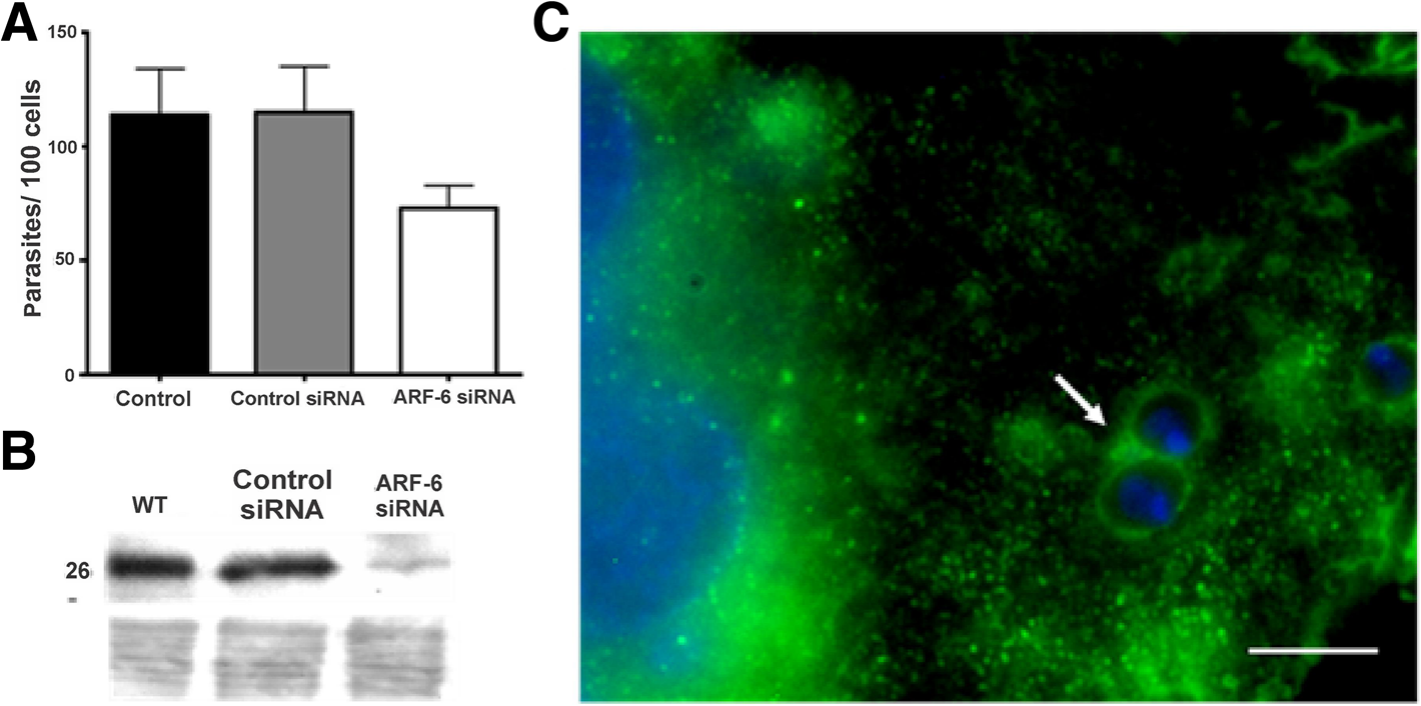
ARF-6 is recruited to *T. cruzi* EA phagosome and its expression is required during host cell invasion. **a:** Wild-type MEF cells were treated with control siRNA, ARF-6 siRNA (Santa Cruz Biotechnology) as previously described (Silva *et al.*, 2009). G-strain EAs were allowed to invade cells for space of one hour. Cells were then washed with PBS, fixed with Bouin and stained with Giemsa. The protocol was similar to the one described in Fig. 1. **b:** Wild-type MEF cell extracts were submitted to Sodium dodecyl sulfate- Polyacrylamide gel electrophoresis (SDS-PAGE) followed by electro-transfer into a nitrocellulose membrane for one hour at 250 mA per cm^2^, incubation with polyclonal antibody anti-ARF-6 [diluted 1:100 in phosphate-buffered saline (PBS)] (Santa Cruz Biotechnology). After washes, membranes were incubated with peroxidase conjugated IgG anti-mouse (diluted 1:5000 in PBS) (Sigma-Aldrich) and developed by chemiluminescence (Silva *et al.*, 2009). **c:** 5x10^5^ wild-type MEF cells/well were transfected with HA-ARF-6 plasmid (a gift from Prof. Dr. Philippe Chavrier, Department of Cell Biology, Research Center, Institut Curie.) and incubated with G-strain EAs for one hour. Cells were then formaldehyde-fixed and incubated with rabbit polyclonal antibody anti-HA (Santa Cruz Biotechnology) [diluted 1:100 in PBS + 0.02 % gelatin + 0.01 % azide (PGN)] followed by AlexaFluor® 488 conjugated IgG anti-rabbit (Invitrogen) (diluted 1:100 in PGN). Arrows indicate EA phagosome enriched in ARF-6. Bar: 10 μm. (p < 0.01)

Annexin A2 knockout cells displayed an important reduction in EA cell invasion. In contrast, lack of Annexin A2 expression favored EA intracellular multiplication (Fig. 1a and b). Furthermore, ARF-6 knock-down fibroblasts also showed significantly lower number of internalized parasites compared to control cells (Fig. 2a and b). Also, our results demonstrated the accumulation of ARF-6 (Fig. 2c) around EA parasitophorous vacuole. Unfortunately, due to the high recovery dynamics of ARF-6 turnover, we could not evaluate the impact of ARF-6 reduced expression over parasite intracellular multiplication using siRNA treatment.

Although not a common property within the annexin family, several annexins have been shown to interact directly with polymerized actin *in vitro*, which correlates with the functions that have been proposed for these annexins in mediating, stabilizing and/or regulating membrane–actin interactions [11]. Annexin A2, both as a monomer or in the heterotetrameric complex with S100A10, was the first annexin shown to be capable of binding to and also bundling actin filaments in a Ca^2+^-dependent manner [11]. Despite its ability to bundle actin filaments, annexin A2 is not found to be associated with prominent actin bundles or cables within cells, such as microvilli or stress fibers. However, more dynamic actin structures, in particular those associated with cellular membranes during phagocytosis, pinocytosis and cell migration contain annexin 2 [11]. Annexin A2 participates in lipid raft formation [12] and is a PIP_2_-interacting protein, thereby explaining its affinity to lipid membranes [13]. Taken together, it has been shown that *T. cruzi* EAs induce host cell calcium signaling [14] and mobilize lipid raft domains during host cell invasion [15].

Authors have shown that Annexin A2 functions as a platform for actin remodeling in the vicinity of dynamic cellular membranes [16]. In this context, lack of Annexin A2 expression may have disorganized the actin cytoskeleton which allowed higher parasite multiplication in knockout cells compared to the wild-type ones. Accordingly, Mott *et al.* (2009) [17] suggested a physical picture in which an intact, stiff, and rapidly remodeling cytoskeleton facilitates early stages of *T. cruzi* invasion and parasite retention, followed by subsequent softening and disassembly of the cytoskeleton to accommodate intracellular replication of parasites. These results pointed to actin cytoskeleton as a potential target for novel therapies in order to restrain parasite multiplication and disease progression.

ARF-6 regulates membrane trafficking and interactions of actin cytoskeleton with the plasma membrane. It is involved in membrane trafficking during receptor-mediated endocytosis, endosomal recycling and exocytosis of secretory granules. It is also implicated in the formation of actin-rich membrane protrusions and ruffles. Trafficking of rafts seems to be a major regulatory pathway by which ARF-6 controls Rac1 activation and cell spreading. Induction of PIP_2_-enriched ruffles and PIP_2_-positive actin-coated vacuoles by ARF-6 leads to a concomitant accumulation of the annexin 2-p11(S100A10) complex [13]. In this sense, we speculate that during EA-host cell interaction Annexin A2 and ARF-6 may act in a convergent manner.

In summary, our findings demonstrated that ARF-6 and Annexin A2 are involved in the invasion of mammalian cells by *T. cruzi* amastigotes. Also, ARF-6 was recruited to the amastigote-containing parasitophorous vacuole-containing amastigote. We postulated that ARF-6 and Annexin A2 participate in the reorganization of the actin cytoskeleton during amastigote cell invasion but the exact mechanism remains to be elucidated.
